# Overhauser Geomagnetic Sensor Based on the Dynamic Nuclear Polarization Effect for Magnetic Prospecting

**DOI:** 10.3390/s16060806

**Published:** 2016-06-01

**Authors:** Jian Ge, Haobin Dong, Huan Liu, Zhiwen Yuan, He Dong, Zhizhuo Zhao, Yonghua Liu, Jun Zhu, Haiyang Zhang

**Affiliations:** 1 School of Automation, China University of Geosciences, Wuhan 430074, China; cekong@cug.edu.cn; 2 Science and Technology on Near-Surface Detection Laboratory, Wuxi 214035, China; zhiwenyuan63@gmail.com (Z.Y.); edmundzhao74@gmail.com (Z.Z.); liucheryl79@gmail.com (Y.L.); zhujun0506@gmail.com (J.Z.); 1987hyzhang@gmail.com (H.Z.); 3 Institute of Geophysics and Geomatics, China University of Geosciences, Wuhan 430074, China; liuhuancug@gmail.com; 4 School of Mechanical Engineering and Electronic Information, China University of Geosciences, Wuhan 430074, China; donghe@2013.cug.edu.cn

**Keywords:** dynamic nuclear polarization effect, Overhauser geomagnetic sensor, free radical, anti-interference, resonant cavity

## Abstract

Based on the dynamic nuclear polarization (DNP) effect, an alternative design of an Overhauser geomagnetic sensor is presented that enhances the proton polarization and increases the amplitude of the free induction decay (FID) signal. The short-pulse method is adopted to rotate the enhanced proton magnetization into the plane of precession to create an FID signal. To reduce the negative effect of the powerful electromagnetic interference, the design of the anti-interference of the pick-up coil is studied. Furthermore, the radio frequency polarization method based on the capacitive-loaded coaxial cavity is proposed to improve the quality factor of the resonant circuit. In addition, a special test instrument is designed that enables the simultaneous testing of the classical proton precession and the Overhauser sensor. Overall, comparison experiments with and without the free radical of the Overhauser sensors show that the DNP effect does effectively improve the amplitude and quality of the FID signal, and the magnetic sensitivity, resolution and range reach to 10 pT/Hz${}^{1/2}$@1 Hz, 0.0023 nT and 20–100 μT, respectively.

## 1. Introduction

The geomagnetic sensor is an essential tool in magnetic prospecting that enables the detection of geological structures and mineral resources (or other objects) through mapping magnetic anomalies produced by rocks and ore bodies [1,2]. The geomagnetic sensor is widely applied to mineral exploration and also has scientific and engineering applications in areas, such as earth and space sciences, military detection, navigation and the non-destructive testing of materials.

For magnetic prospecting in the field, the sensors require high sensitivity and a wide range (20,000–100,000 nT). Especially, the small dimension and low power consumption are also very important in the walking measurement. At present, the main geomagnetic sensors for the absolute measurements of the total magnetic intensity include optically-pumped and classical proton precession (CPP) sensors [3]. The optically-pumped sensor provides high sensitivity, continuous output and fast sampling. Note that it requires a light source and a lens system, and the alkali vapor sensor also needs to be preheated [4,5,6]. The optically-pumped sensor is excellent for airborne surveys and other applications where high tracking speed is required. The CPP sensor is still the more common geomagnetic sensor for portable measurements [7]. However, as the energy splitting and the gyromagnetic ratio of the proton are quite small, the initial amplitude of the free induction decay (FID) signal that the sensor outputs is only about 0.5 μV; even the most excellent weak signal processing circuit could not effectively improve the signal to noise ratio (SNR) to a satisfactory amount. Moreover, before a magnetic field measurement, a strong direct current (1–5 A) that lasts several seconds (1–3 s) is needed for pre-polarization; this significantly increases the power consumption [8,9]. Consequently, the magnetic sensitivity and power consumption of the CPP sensor remain unsatisfactory.

With the deficiencies of the CPP sensor in mind, the FID signal enhancement method based on the dynamic nuclear polarization (DNP) effect is adopted in this study. By using the cross-relaxation between proton and electron spins, the electron polarization can be transferred to the proton polarization during irradiation at a certain electron resonant frequency, and thereby, the amplitude of the FID signal can be improved [10,11]. This method not only improves the magnetic sensitivity, but also can effectively reduce the power consumption using radio frequency (RF) irradiation.

The DNP effect was first discovered by Overhauser in 1953 [12]. Slichter *et al*. gave the first demonstration of the DNP effect. Afterwards, Abragam *et al*. further investigated the DNP effect in nonmetals and paramagnetic liquids [13]. At present, the DNP effect is widely used in NMR experiments, with a view toward being applied in medicine and chemistry. Furthermore, several researchers have analyzed the magnetic field sensor based on the DNP effect using different liquid samples. Duret *et al*. designed an Overhauser sensor based on the self-oscillating method for the Oersted satellite [14,15,16]. The sensor is still operational; however, its design was not described in great detail. This self-oscillating sensor is composed of two flasks, which contain a pair of solvents, and requires an additional feedback circuit to feed the proton precession signal back to the coils to produce the continuous polarization field; hence, the major advantage over this method is great speed in following magnetic field changes. This may require a more complex design, a larger volume and higher power consumption [14]. Using a brief diagram, Sapunov [17] and Hrvoic [18,19] simply introduced the differences between the CPP sensor and the Overhauser sensor, and preliminarily analyzed the absolute and random errors, thermal effects and calibration. However, the design of the Overhauser sensor was not mentioned. At present, although there are some commercial Overhauser sensors, few published papers and patents involve any detailed design [20].

Based on the previously-mentioned work, we conducted a detailed investigation of the Overhauser geomagnetic sensor for magnetic prospecting based on the short-pulse method and proposed several improvements. The design of the anti-interference of the pick-up coil is studied to reduce the negative effect of the powerful electromagnetic interference (EMI). To achieve the DNP effect, a sample containing nitroxide and organic solvent was compounded. For the ohmic, dielectric and radiation loss of a conventional LCresonant circuit, a coaxial resonant cavity is proposed to achieve the RF polarization of the sample. Finally, a special test instrument is designed to test the enhancement of the FID signal and to measure the main specifications of this sensor.

## 2. FID Signal Enhancement Method Based on the DNP Effect

The amplitude of the FID signal output from the CPP sensor is [7]:

(1) $$V\left(t\right)=c\times M_{p}\times \omega_{p}\times sin^{2}\theta \times e^{-t/T_{2}}sin\omega_{p}t$$

where *c* is a constant, $T_{2}$ is the transverse relaxation time of the sample rich in protons, *θ* is the angle between the coil axis and measured magnetic field, $M_{p}$ is the proton magnetization and $\omega_{p}$ is the angular frequency of the proton precession. Given the readings of the frequency meter, the geomagnetic field $B_{0}$ could be calculated using [7],

(2) $$\omega_{p}=\gamma_{p}B_{0}$$

where $\gamma_{p}$ is the proton gyromagnetic ratio.

According to Equations (1) and (2), when $M_{p}$ is enhanced, the amplitude of the FID signal is increased; furthermore, the precision of the frequency meter also increases; hence, the magnetic sensitivity of the sensor is improved. As $M_{p}$ is proportional to the expectation value of the proton polarization, the DNP effect can improve the sensitivity of the CPP sensor through the enhancement of the proton polarization.

The achievement paths of the DNP effect include four distinct mechanisms [21]: the solid effect, which has limited experimental use; the Overhauser effect, which is the only one that has an appreciable effect for liquids; and the cross effect/thermal mixing, which is the predominant effect for contemporary solid-state and dissolution DNP. According to Equation (1), $T_{2}$ determines the time interval available for the measurements of the frequency of the FID signal. In liquids, $T_{2}$ may reach several seconds, whereas in solids, $T_{2}$ is only milliseconds. Therefore, we focus on DNP in liquid free radicals, and we present only a summary of the Overhauser effect as it relates to this type of sensor.

**Figure 1:**
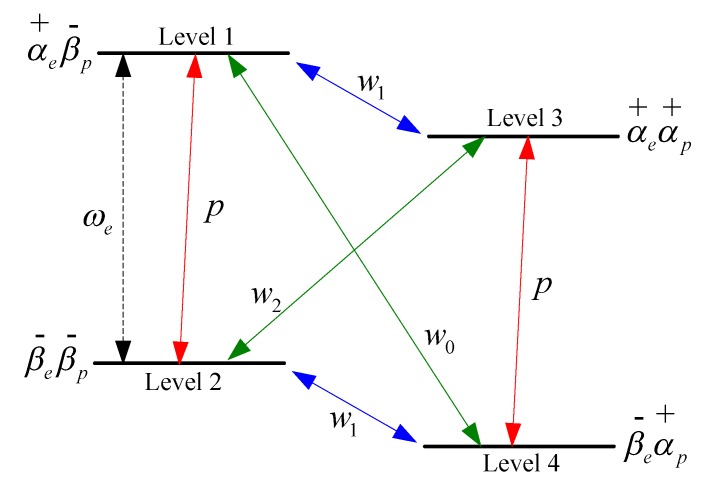
Four-level energy diagram for an electron spin *S* = 1/2 coupled to a ${}^{1}$H proton spin *I* = 1/2.

As shown in Figure 1, the Overhauser effect for two spins (an electron spin *S* = 1/2 and ${}^{1}$H proton spin *I* = 1/2) is typically described by a four-level diagram. Here, *p* is the electron spin relaxation rate, $w_{1}$ the proton spin relaxation rate, $w_{2}$ the double quantum relaxation rate and $w_{0}$ the zero quantum relaxation rate. The basic theory of the Overhauser enhancement is as follows: an RF alternating field (of angular frequency $\omega_{e}$) saturates the electron spin relaxation, which creates a non-equilibrium population distribution of the electron spins. The electron-proton cross-relaxation then transfers the electron spin polarization from the electron spin to the proton spin. A non-equilibrium proton spin polarization is thereby achieved, implying that the proton magnetization is also enhanced [22,23,24]. According to the above analyses, the sample in the Overhauser sensor must simultaneously maintain a stable electron-proton spin system. Typically, an organic solvent rich in protons and a free radical are used to provide stable proton and electron spin systems, respectively.

The enhancement of the proton polarization, *E*, is defined as:

(3) $$E=\frac{\langle I_{z}\rangle }{\langle I_{o}\rangle }\approx 1-\rho fs\frac{\gamma_{e}}{\gamma_{p}}$$

where $\langle I_{z}\rangle$ is the expectation value of the DNP, $\langle I_{o}\rangle$ is its thermal equilibrium value [25], $\gamma_{e}$ is the electron gyromagnetic ratio and *ρ* and *f* are, respectively:

(4) $$\rho =\frac{w_{2}-w_{0}}{w_{2}+2w_{1}+w_{0}}$$

(5) $$f=1-\frac{T_{1}}{T_{10}}$$

The coupling factor *ρ* is understood as the ratio of the electron-proton spin cross-relaxation rate ($w_{2}-w_{0}$) and the proton spin relaxation rate due to the electrons ($w_{2}+2w_{1}+w_{0}$) [26]. *ρ* expresses the efficiency of coupling between the electron and proton spins and ranges from −1 (pure scalar coupling) to 0.5 (pure dipolar coupling) [27]. For free radicals dissolved in solution, the coupling of the electron spins to solvent protons can be either scalar or dipolar [28], and hence, *ρ* can be safely assumed to be one.

The leakage factor *f* relates to the electron’s ability to relax the proton spin and can be expressed in terms of the longitudinal relaxation times $T_{1}$ and $T_{10}$ of the solvent in the presence and absence of the free radical, respectively. A leakage factor of one implies that all relaxations of the protons are caused by electrons, whereas a leakage factor of zero occurs when all relaxations of the protons are through other sources [21].

The saturation factor *s* is the degree of saturation of ESR, and varies from 0 to 1, depending on the power of the applied RF electromagnetic field [21].

Ideally, $s_{max}=1$ and $f_{max}=1$ can be safely assumed; therefore, $E_{max}=1+\gamma_{e}/\gamma_{p}$ = 660 [27]. In other words, the amplitude of the FID signal can in theory be increased by 660 times. However, the theoretical increase $E_{max}$ cannot be achieved for several reasons, one being that other paths of relaxation are present.

## 3. Design of the Overhauser Sensor

In the CPP sensor, the proton polarization and the deflection into the plane of precession of the enhanced proton magnetization is achieved simultaneously [7]. By contrast, for the Overhauser sensor, the DNP effect only enhances the proton polarization. Consequently, the enhanced proton magnetization needs to be further rotated into the plane of precession to create an FID signal. Our study adopts the short pulse method to achieve this rotation. Compared to the Overhauser sensor based on the self-oscillating method, it only needs a flask of a sample to generate a positive or negative polarization.

**Figure 2:**
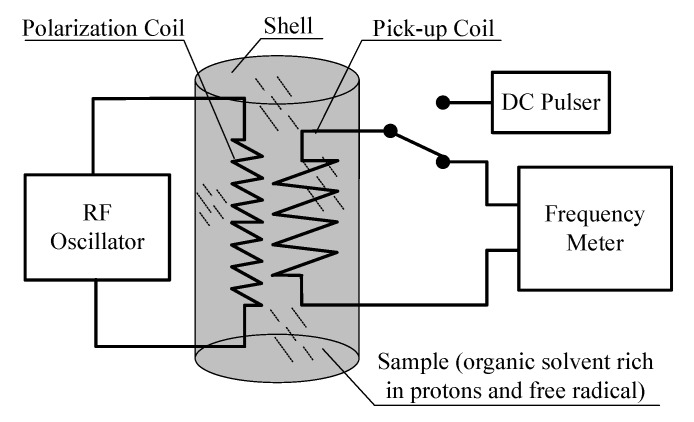
Scheme of the Overhauser geomagnetic sensor.

Accordingly, as shown in Figure 2, the scheme of the Overhauser sensor based on the DNP effect is designed with a polarization coil, a sample (proton-rich organic solvent and free radical) and a pick-up coil.

**Figure 3:**
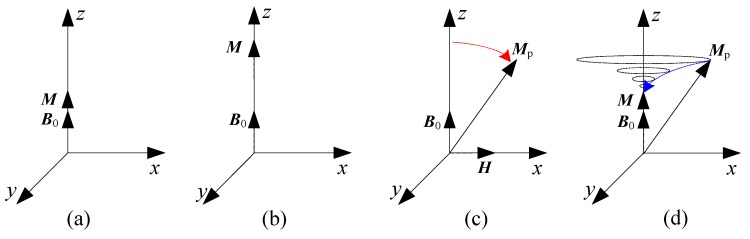
Enhancement and deflection of proton magnetization. (**a**) Initial state; (**b**) enhancement; (**c**) deflection; (**d**) rotating.

**Figure 4:**
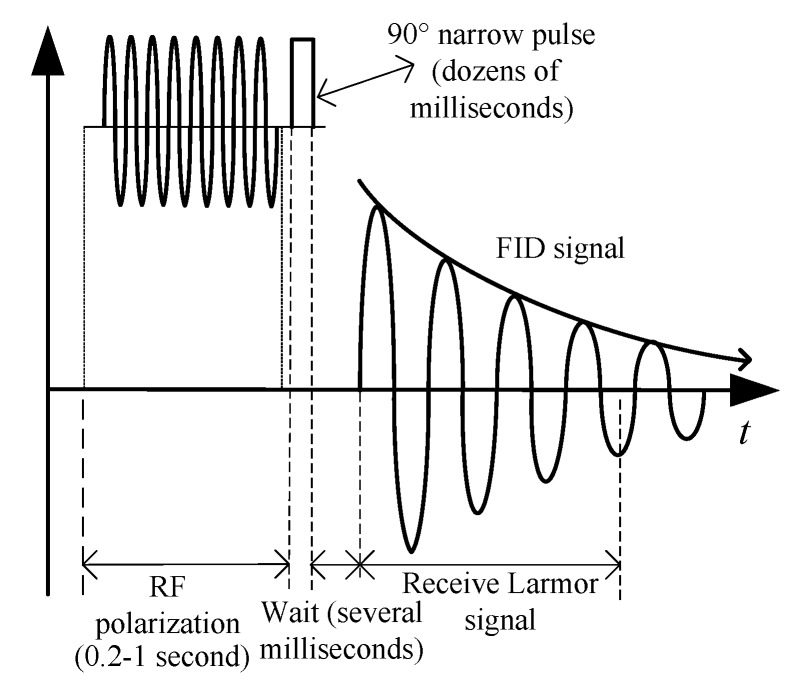
Workflow of the Overhauser sensor.

Figure 3 shows the enhancement and deflection of the proton magnetization in the sensor. As shown in Figure 3a,b, the sensor realizes the DNP effect using an alternating field generated by an RF oscillator and polarization coil; thereby, the proton magnetization ***M*** along the external magnetic field $B_{0}$ is enhanced. Next, as shown in Figure 3c, after full proton polarization, the RF oscillator is switched off, and a $90^{\circ }$ narrow pulse (the width is only dozens of milliseconds) is injected into the pick-up coil to generate a DC deflection field ***H***, which deflects the enhanced proton magnetization into the plane of precession and establishes a new proton magnetization $M_{p}$. As shown in Figure 3d, after disconnecting the pulser, as with the CPP sensor, $M_{p}$ would rotate around the external magnetic field $B_{0}$ and restore to the state as shown in Figure 3a. In the backhaul, an FID signal is induced in the pick-up coil; its mathematical expression is given in Equation (1). The workflow associated with this process is shown in Figure 4.

### 3.1. Design of Anti-Interference

The initial amplitude of the FID signal received by the Overhauser sensor is only of the order of a microvolt. Hence, in the outdoor measurements, the sensor is easily affected by a variety of powerful EMI sources, such as power lines and broadcast communications. Indeed, the FID signal can be seriously swamped entirely. Consequently, the anti-interference capability is particularly paramount for the Overhauser sensor to function.

**Figure 5:**
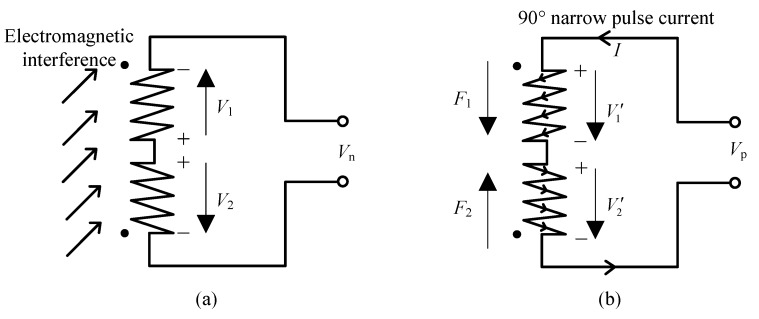
Anti-interference method of the differential dual-coil structure: (**a**) interferences cancel each other; (**b**) receiving Larmor signals.

Normally, a pick-up coil based on a uni-coil structure can be used to detect the FID signal. While simple to install and small in size, this uni-coil nonetheless receives not only the FID signal, but the external EMI. This structure has poor anti-interference capability. Therefore, a differential dual-coil structure [15,16] comprising two series-opposing coils is applied as shown in Figure 5a. As the size of this sensor is small, the external EMI is assumed to be uniformly distributed. According to Faraday’s law and Lenz’s law, when the electrical and geometrical parameters of the two opposing coils, such as inductance, resistance and dimension, are the same, the absolute values of the two EMI voltages (denoted as $V_{1}$ and $V_{2}$) induced in these two coils are equal, but opposite in direction. As a result, the induced voltage $V_{n}$ of the external EMI is canceled,

(6) $$V_{n}=V_{1}+\left(-V_{2}\right)\approx 0$$

According to the workflow of the Overhauser sensor, the RF polarization field is used to generate the DNP effect, and then, the directions of all of the proton magnetic moments are almost the same. As shown in Figure 5b, after RF polarization, when there is a $90^{\circ }$ narrow pulse current, which is injected into the pair of coils, two opposing deflection magnetic fields (denoted as $F_{1}$ and $F_{2}$) are generated. Hence, the respective initial phases of the proton magnetic moments near the two coils differ by $180^{\circ }$, forcing the initial phases of the two voltages (denoted as $V_{1}^{{}^{\prime }}$ and $V_{2}^{{}^{\prime }}$) induced in the two coils also to differ by $180^{\circ }$. Moreover, as two coils are connected in series and opposing, the directions of the two voltages are the same, and their absolute values are equal. Consequently, the induced voltage $V_{p}$ of the FID signal is:

(7) $$V_{p}=V_{1}^{{}^{\prime }}+V_{2}^{{}^{\prime }}\approx 2V_{1}^{{}^{\prime }}\approx 2V_{2}^{{}^{\prime }}$$

Therefore, according to Equations (6) and (7), the FID signal received by the entire pick-up coil is:

(8) $$V=V_{p}+V_{n}\approx V_{p}\approx 2V_{1}^{{}^{\prime }}$$

**Figure 6:**
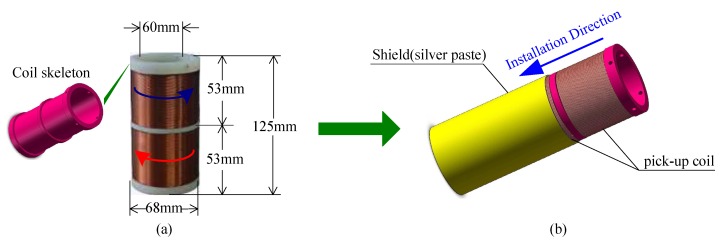
Photograph of the receiving coil. (**a**) Two series-opposing coils; (**b**) the installation of the coils and shield.

Note that current manufacturing techniques cannot ensure that the electrical and geometrical parameters of the two coils are identical, and it is impossible that the external EMI nearby a sensor is uniformly distributed with certainty. Notwithstanding these limitations, this differential structure still is able to suppress much of the EMI and, consequently, to improve the anti-interference capability of the sensor. In addition, it should be noted that there are distributed capacitances between the layers and turns of the coils, which are difficult to quantify, and hence, the accuracy of the natural frequency of the resonant circuit in the instrument inevitably suffers. However, the series of sensor coils could effectively reduce the distributed capacitance. In Figure 6a, the two series-opposing coils are shown as wound in two grooves of the coil skeleton; the inductance of the pick-up coil is about 30 mH.

Another effective anti-interference method is EMI shielding. Typically, copper foil and a copper network are used for EMI shielding in the magnetic sensor. However, its shielding effectiveness is not high (generally 40–60 dB). To resolve this problem, conductive silver paste as the coating material is used for shielding and can enhance the shielding effectiveness to 80 dB. As shown in Figure 6b, the pick-up coil and shield comprise the pick-up components. Simultaneously, aside from the coppery shielding shell coating the sensor interface, the pick-up coil and instrument are connected through a shielded audio cable, thereby minimizing interference during signal transmission.

### 3.2. Design of Resonant Cavity

The RF polarization of the sample is the key in producing the DNP effect. In considering the stability requirement for the geomagnetic sensor, 4-Oxo-TEMPOand dimethyl ether as the free radical and organic solvent, respectively, were chosen. Moreover, the dimethyl ether adds an additional benefit in guaranteeing the chemical stability of 4-Oxo-TEMPO. It is noteworthy that the electrons exhibit strong scalar coupling with the nitrogen nuclei in the molecules of 4-Oxo-TEMPO. The effect of this interaction is that the electrons dwell in a local magnetic field of nitrogen nuclei of about 2.14 mT [28]. Consequently, the frequency of the RF polarization is no longer 1.4 MHz (in the geomagnetic field of 50,000 nT), but more than 60 MHz. From Figure 2, the 60-MHz RF polarization component actually consists of two parts: the oscillator in the instrument and the polarization coil in the sensor. The oscillator provides a 60-MHz alternating signal, which is then injected into the polarization coil to produce an RF field. The polarization enhancement is greater if the SNR of the RF signal is higher. Conventionally, a resonant circuit is used to further improve the quality of the RF signal output from the RF oscillator. An LC resonant circuit consisting of an RF coil and capacitors could be used. However, it is not suitable for an Overhauser sensor for two reasons:

(1) Compared to achieving low-frequency resonance, achieving high-frequency resonance requires the inductance and capacitance of the circuit to be reduced, which would rapidly decrease the component size and, moreover, reduce the capacity and mechanical strength of the resonant circuit.
(2) The skin effect of electromagnetic waves would be intensified with the increase in frequency, and then, the ohmic, dielectric and radiation loss would increase significantly, leading to a considerable decrease in quality factor Q of the resonant circuit.

**Figure 7:**
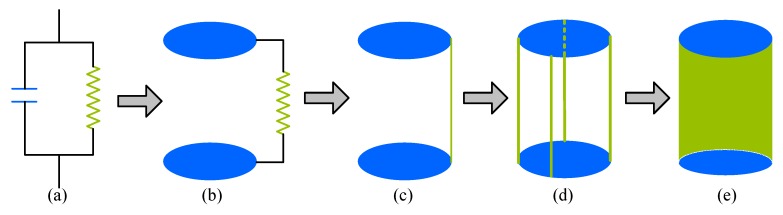
Evolution of the resonant cavity. (**a**) LC resonant circuit; (**b**) spacing the two plates of the capacitor; (**c**) reducing the number of turns of the inductor; (**d**) several parallel straight wires; (**e**) a closed cavity.

To target these problems, we opted for the resonant cavity to achieve a high Q resonant circuit. To increase the resonant frequency, the capacitance and inductance must be small. As shown in Figure 7, spacing the two plates of the capacitor further apart would reduce the capacitance, and reducing the number of turns of the inductor and even straightening the inductance coil would decrease the inductance. Moreover, several parallel straight wires would further decrease the inductance, and therefore, a closed resonant cavity would form as soon as the number of straight wires becomes sufficiently large.

In the resonant cavity, as the electromagnetic waves reflect back and forth along the cavity wall and establish standing waves, the electromagnetic field becomes confined within the cavity. There is a phase difference of $90^{\circ }$ between the electric field and the magnetic field; hence the oscillation of the electromagnetic wave in the resonant cavity is similar to that in an LC resonant circuit. Both of them involve the electromagnetic energy transfer between electric and magnetic fields. However, the quality factor of the resonant cavity is much higher than that for the LC resonant circuit. The decrease in internal resistance leads to a great reduction in ohmic loss. Moreover, the cavity has advantages in avoiding radiation loss and storing energy, which could greatly reduce the crosstalk of the RF signal between the sensors during magnetic gradient measurements.

Typical resonant cavities include the coaxial, rectangular and cylindrical resonant cavity. For the rectangular and the cylindrical resonant cavity, the wavelength of the resonant signal is [29,30]:

(9) $$\lambda_{0}=\frac{1}{\sqrt{{\left(\frac{1}{\lambda_{c}}\right)}^{2}+{\left(\frac{p}{2l}\right)}^{2}}}$$

where $\lambda_{c}$ is the wavelength corresponding to the resonant frequency, $\lambda_{0}$ is the wavelength corresponding to the cut-off frequency of the resonant cavity, *p* is a positive integer and *l* is the distance between the two end-faces of the resonant cavity. According to Equation (9),

(10) $$l=\frac{p\lambda_{0}\lambda_{c}}{2\sqrt{\lambda_{c}^{2}-\lambda_{0}^{2}}}$$

Because the 60-MHz RF field corresponds to the wavelength of 5 m, *l* is at least greater than half of $\lambda_{0}$ (2.5 m), according to the above equation, which is clearly too long for a portable geomagnetic sensor.

**Figure 8:**
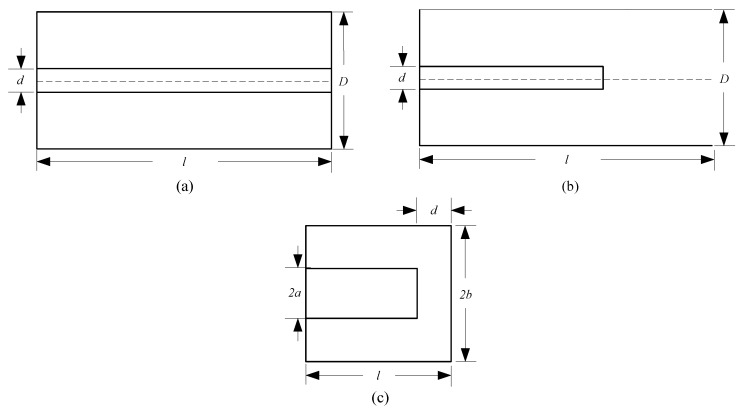
Scheme of the coaxial cavity: (**a**) λ/2, (**b**) λ/4 and (**c**) capacitive-loaded coaxial cavity.

Coaxial resonant cavities include λ/2, λ/4 and capacitive-loaded coaxial cavities. As shown in Figure 8a,b, to achieve resonance, λ/2, λ/4 coaxial cavities require a distance between the two end-faces to be an integer multiple of λ/4, respectively [31]; that is, the cavity lengths are at least 2.5 m and 1.25 m, respectively, which again do not meet the size requirement. Hence, we chose the capacitive-loaded coaxial cavity for which the distance between the two end-faces can be reduced through the loaded capacitor. The cavity could then be downsized. Viewing the schematic of the cavity as shown in Figure 8c, there is an equivalent loaded capacitor between the end-face and the short-circuit plate, which could be regarded as a plate capacitor. Moreover, from a theoretical derivation, the length *l* of the cavity can be shortened if the capacitance is increased.

**Figure 9:**
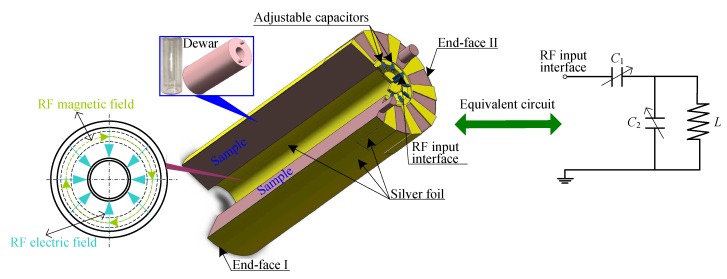
Scheme of the capacitive-loaded coaxial cavity in the Overhauser sensor.

A capacitive-loaded coaxial cavity is designed as shown in Figure 9. To further reduce the ohmic loss of the cavity as much as possible, a silver foil is used as a conducting shell for the cavity. As the energy of the resonant cavity is mainly concentrated in the cavity, the cavity is directly mounted onto the surface of a cylindrical Dewar flask filled with the sample, so as to fully polarize the sample. In addition, as the free radical places high demand on the accuracy of the RF frequency, several adjustable capacitors are placed at the input interface of the resonant cavity to adjust the resonant frequency and quality factor. The RF magnetic field and the electric field are shown on the left side in Figure 9.

**Figure 10:**
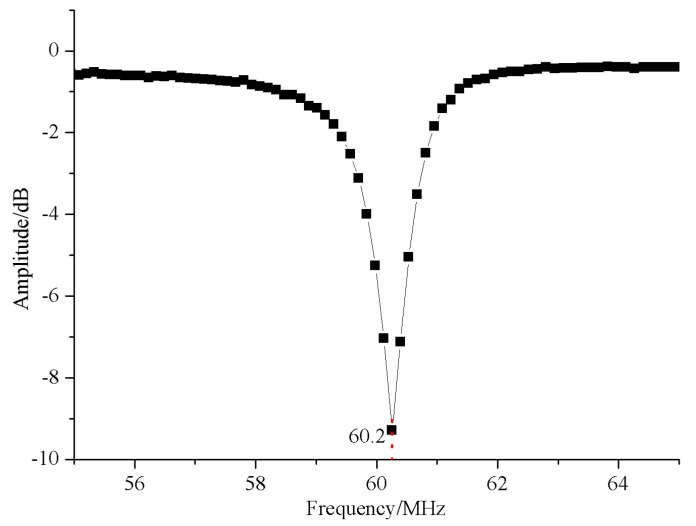
Frequency characteristics of the capacitive-loaded coaxial cavity.

On the right side in Figure 9, the resonant cavity is equivalent to a series resonant circuit. With the designed cavity (L≈ 150 nH, $C_{1}\approx$ 6.5 pF, $C_{2}\approx$ 40 pF), the frequency characteristics of the resonant cavity (Figure 10) yield a resonant frequency of about 60.2 MHz.

**Figure 11:**
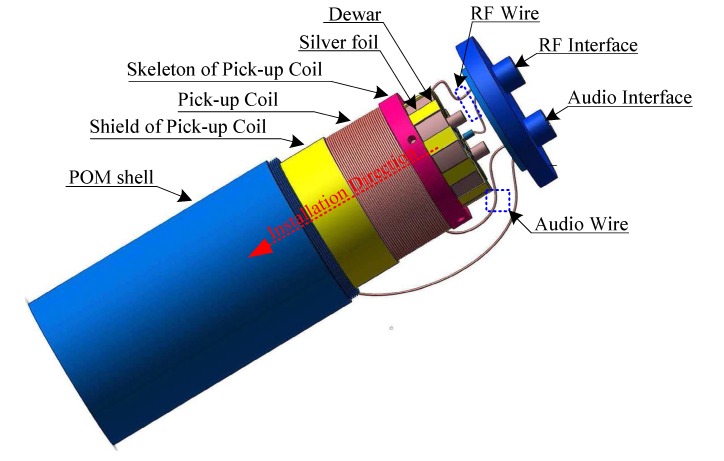
Composition of the Overhauser sensor.

With the capacitive-loaded coaxial cavity and differential dual-coil, the completed Overhauser sensor as shown in Figure 11 was fit into a cylindrical form of 140 mm in length and 70 mm in diameter. To fully induce an FID signal, the resonant cavity was inserted in the cavity of the pick-up component (*i.e*., pick-up coil and shield), and both components were then installed in a polyoxymethylene shell. Through the RF wire and interface, an RF signal from the oscillator of the instrument is applied to the resonant cavity. Furthermore, the pick-up coil is connected to the frequency meter by means of the audio wire and interface.

## 4. Design of the Test Instrument

**Figure 12:**
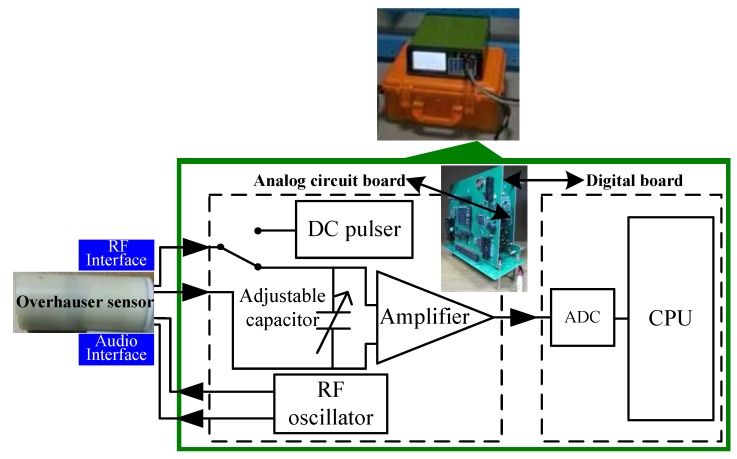
Block diagram of the Overhauser sensor test instrument.

To test the Overhauser sensor, a special test instrument (Figure 12) was designed based on the characteristics of the FID signal [32]. This instrument consists of an RF oscillator, DC pulser, series resonant circuit, amplifier, analog-to-digital converter (ADC) and central processing unit. Note that if the RF oscillator is removed, this instrument can be used to test also the CPP sensor.

As the geomagnetic field ranges from 20,000 nT–100,000 nT, the frequency of the FID signal must range from 850–4500 Hz. Before a frequency measurement, the weak FID signal must be amplified and processed, and the conventional method is to use a broadband amplifier. Nevertheless, this method amplifies not only the FID signal, but also noise. Even with the best broadband amplifier, the requirements for the following frequency meter cannot be met. In general, the bandwidth of the amplifier could be limited to improve the SNR. However, this would conflict with the wider bandwidth of the FID signal. In response to this contradiction, we adopted the adjustable series resonant circuit, which consists of a pick-up coil and several adjustable capacitors to achieve frequency-selective amplification within a narrow band, as well as a frequency coverage of 850–4500 Hz. After passing through the series resonant circuit, the SNR of the FID signal is significantly increased. By using a broadband amplifier with a bandwidth of 850–4500 Hz and a gain of hundreds of thousands, the signal quality could be further improved.

From Equation (2), the precision of the frequency measurement of the FID signal directly determines the sensitivity of the magnetic field measurement. The conventional frequency measurement methods are based on a hardware scheme, which employs a comparator to shape the FID signal into a square wave and then measures the frequency. There are many common frequency measurement methods for the square wave, such as the direct frequency measurement method, the period measurement method and the multi-cycle synchronous method. The essence of these methods involves taking advantage of a standard timer or counter to realize the frequency measurement; the longer the measurement time lasts, the higher the precision is. However, in the later period of the exponentially decaying FID signal, when the SNR considerably decreases, the zero-crossing interference and other noises would lead to shaping errors of the comparator, which would bring new counting errors. In response to this problem, we developed a digital frequency measurement scheme based on the spectrum zoom algorithm. This method uses a high-precision ADC and the fast Fourier transformation algorithm to obtain a rough estimate of the frequency of the FID signal. The chirp z transform algorithm is then applied to amplify the narrowband spectrum nearby this frequency estimate and, therefore, to achieve a high-precision frequency measurement that avoids the counting errors of conventional methods.

## 5. Experiments

### 5.1. FID Signal Comparison

**Figure 13:**
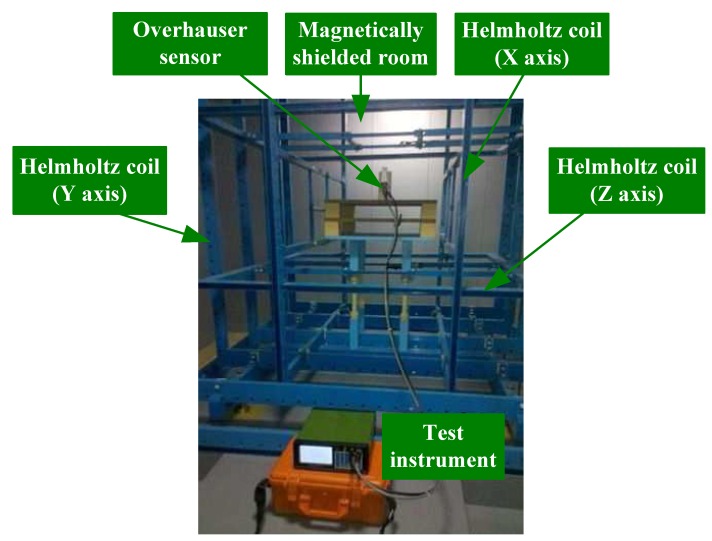
Artificial magnetic field-generating space.

An FID signal comparison is used to verify the enhancement of the proton polarization in the DNP effect. The experiment was assembled in an artificial magnetic field-generating space shown in Figure 13, which consists of a magnetic field generator (including a high-precision constant current source, a standard magnetometer and a three-axis Helmholtz coil system) and a magnetically-shielded room. Moreover, located far away from all electromagnetic sources, this space not only shielded the external EMI, but also canceled the local geomagnetic field. It produced a stable artificial magnetic field of high precision in all directions.

**Figure 14:**
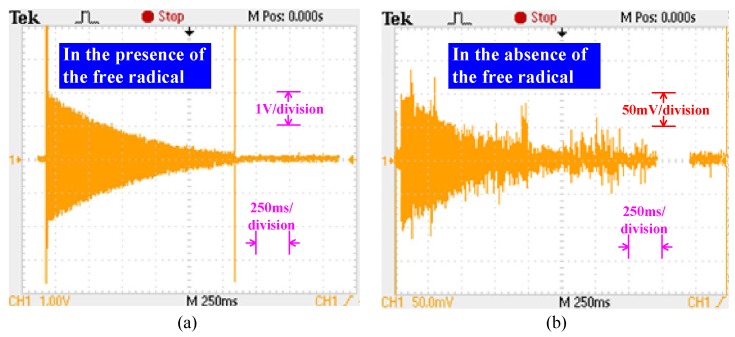
Comparison results of the free induction decay (FID) signal. (**a**) In the presence of the free radical; (**b**) in the absence of the free radical.

Figure 14 shows a comparison of the results for the amplitude of the FID signal in the presence and absence of the free radical with the same sensor and test instruments described above. The initial amplitude of the FID signal under two different conditions was 4 V and 200 mV, respectively. The 20-fold gain indicates that the DNP effect significantly improves the amplitude and quality of the FID signal under the action of the free radical. Note that, because the comparison experiments adopted the same polarization sequence based on the short-pulse method, the 20-fold gain could not actually be described as a proton polarization enhancement *E* of the dynamic nuclear polarization state relative to the thermal equilibrium state. In addition, the width of the short pulse is only dozens of milliseconds. The initial amplitude of the FID signal of the Overhauser sensor without the free radical is well below that of the CPP sensor based on direct current pre-polarization.

### 5.2. Main Specifications

In general, for the NMR magnetic sensors (e.g., optically pumped, CPP, Overhauser sensors), the main specifications include sensitivity, resolution and range [15,16]. To better reflect the performance of the Overhauser sensor, the study used the most popular commercial CPP sensor GSM-19T and the test instruments described above to implement a contrast test. In a preemptive explanation, although the amplitude of the FID signal from the Overhauser sensor is superior to that of the CPP sensor, all FID signal outputs from both sensors are of the order of microvolts, and the signal bandwidth ranges from 850–4500 Hz. Consequently, the test instruments (consisting of an amplifier and a frequency meter) for the two sensors can be exactly the same (the RF oscillator is no longer needed when testing the CPP sensor). This eliminates the effect of different test instruments and provides a better comparison of the two sensors.

**Figure 15:**
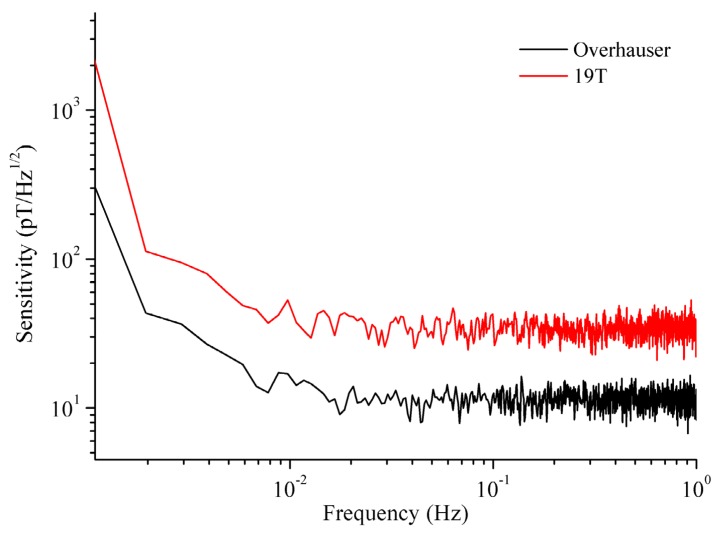
Sensitivities of our sensor and GSM-19T.

For the NMR magnetic sensors, the sensitivity conventionally can be defined as the larger of either the resolution or the noise [33]. As the sensitivity and resolution actually depend on the noise, the noise spectral density in terms of pT/Hz${}^{1/2}$ is usually used to express the sensitivity following the usual practice. The sensitivity experiment was performed at Wuhan National Observation and Research Station of Gravity and Solid Tide, where background field noise can be guaranteed to be lower than 1 pT/Hz${}^{1/2}$@1 Hz. The experimental results are shown in Figure 15; the 10 pT/Hz${}^{1/2}$@1 Hz sensitivity of the Overhauser sensor is lower than 30 pT/Hz${}^{1/2}$@1 Hz for the GSM-19T sensor.

The range experiment was still-tested in the artificial magnetic field generating space (Figure 13). According to Table 1, the range of the Overhauser sensor is from 20,000 nT–100,000 nT. In addition, the 0.2 nT uncertainty (standard deviation) of the Overhauser sensor is lower than the 1.9 nT for the GSM-19T sensor over the range of geomagnetic fields. Moreover, the results also show that the uncertainty increases with the declination of the external magnetic field. From Equations (1) and (2), the initial amplitude of the FID signal is proportional to the precession frequency and external magnetic field; hence, the performance of the frequency meter may decline with decreasing external magnetic field, which finally leads to an increase in uncertainty.

For the Overhauser and CPP sensors, the resolution is the minimum step of the frequency meter used to measure the precession frequency and its conversion into an equivalent magnetic field. Therefore, the resolution is actually determined by the frequency meter according to Equation (2). Generally, in the magnetometer design, the equivalent magnetic resolution is substantially higher (an order of magnitude) than the ultimate sensitivity to avoid a contribution of the counter to the overall noise of the system [34]. The experiment was conducted at Hubei Province Electronic Information Product Quality Supervision and Inspection Institute. The result indicates that the resolution of the frequency meter is 0.0001 Hz, implying that the Overhauser sensor has a resolution of 0.0023 nT.

Moreover, the power consumption of our Overhauser sensor is about 1.9 W, which is lower than the 7 W for the GSM-19T based on the direct current pre-polarization. This would extend the operating hours of the battery in walking measurements.

### 5.3. Field Test

**Figure 16:**
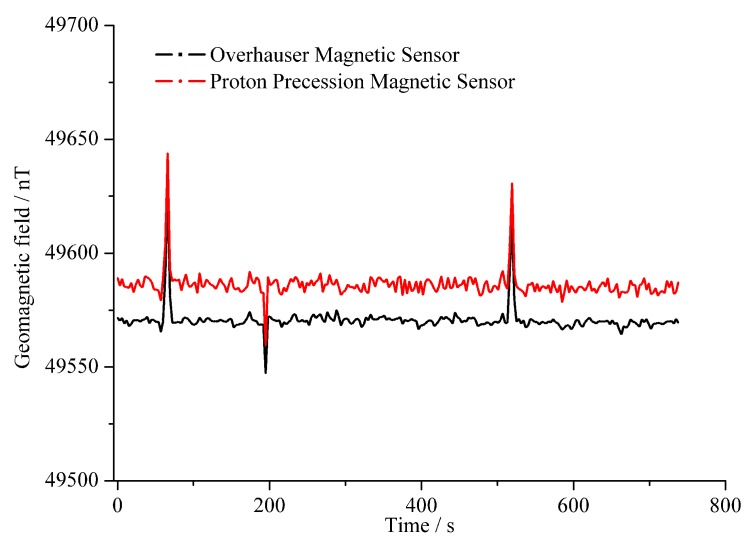
Contrast results of the geomagnetic field measurement between two sensors.

To further verify the performance of the Overhauser sensor, a comparative test of a geomagnetic field measurement was implemented for the Overhauser and GSM-19T sensors in the field. The test environment was far from EMI sources, and the two sensors were separated by 5 m to prevent mutual interference. Moreover, we started up two sensors simultaneously to avoid the influences from geomagnetic diurnal variations. As shown in Figure 16, the results of the comparison indicate that:

(1) The trends in the geomagnetic field measurements from the two sensors are basically coincident, although the two curves did not overlap. The baseline difference is about 15 nT. The main cause is the magnetic field gradients at the locations of the sensors. Despite being 5 m apart, the Earth’s magnetic field gradient can be ignored. In contrast, with the limitations in test conditions, the fixed magnetic anomaly generated by buildings, pipelines and roads nearby the sensors also can give rise to a magnetic field gradient. However, such magnetic anomalies are different from random EMI and do not affect a comparison of the two sensors.
(2) In the field test, to verify the detection capability of the magnetic anomaly of the two sensors, a bicycle was ridden nearby the sensors at 70, 200 and 520 s to generate an artificial magnetic anomaly. The test results show that both of the sensors were able to detect a magnetic anomaly of +30 nT, −10 nT and +20 nT, respectively, which indicated a normal detection capability for the Overhauser sensor.

## 6. Conclusions

To solve the deficiencies of the CPP sensor, our study focused on the DNP effect to further develop an Overhauser geomagnetic sensor for magnetic prospecting. To test the FID signal and performance in magnetic field measurements, a special test instrument was designed that could test both the CPP sensor and the Overhauser sensor. In a comparison of the FID signals in the presence and the absence of the free radical, a 20-fold gain indicated that the DNP effect significantly improves the amplitude and quality of the FID signal under the action of free radicals. Moreover, the 10 pT/Hz${}^{1/2}$@1 Hz sensitivity for the Overhauser sensor is lower than the 30 pT/Hz${}^{1/2}$@1 Hz for the CPP sensor; the resolution and range of the Overhauser sensor are 0.002 nT and 20 μT–100 μT, respectively; the 0.2 nT uncertainty of the Overhauser sensor is lower than the 1.9 nT of the CPP sensor over the range of the geomagnetic field. In addition, the sensor size is 140 mm in length and 70 mm in diameter.

## Figures and Tables

**Table 1:** Results of range and uncertainty (unit: nT).

Artificial Magnetic Field	Average		Uncertainty	
	Overhauser	GSM-19T	Overhauser	GSM-19T
20,112.4	20,112.52	20,113.43	0.2	1.9
49,900.3	49,900.35	49,901.12	0.1	1.1
100,150.7	100,150.78	100,151.15	0.1	0.8
